# SPEAR: Systematic ProtEin AnnotatoR

**DOI:** 10.1093/bioinformatics/btac391

**Published:** 2022-06-13

**Authors:** Matthew Crown, Natália Teruel, Rafael Najmanovich, Matthew Bashton

**Affiliations:** Hub for Biotechnology in the Built Environment, Department of Applied Sciences, Faculty of Health and Life Sciences, Northumbria University, Newcastle upon Tyne NE1 8ST, UK; Department of Pharmacology and Physiology, Université de Montréal, Montreal, QC H3T 1J4, Canada; Department of Pharmacology and Physiology, Université de Montréal, Montreal, QC H3T 1J4, Canada; Hub for Biotechnology in the Built Environment, Department of Applied Sciences, Faculty of Health and Life Sciences, Northumbria University, Newcastle upon Tyne NE1 8ST, UK

## Abstract

**Summary:**

We present Systematic ProtEin AnnotatoR (SPEAR), a lightweight and rapid SARS-CoV-2 variant annotation and scoring tool, for identifying mutations contributing to potential immune escape and transmissibility (ACE2 binding) at point of sequencing. SPEAR can be used in the field to evaluate genomic surveillance results in real time and features a powerful interactive data visualization report.

**Availability and implementation:**

SPEAR and documentation are freely available on GitHub: https://github.com/m-crown/SPEAR and are implemented in Python and installable via Conda environment.

**Supplementary information:**

Supplementary data are available at *Bioinformatics* online.

## 1 Introduction

The SARS-CoV-2 virus caused a global pandemic with >5.8 million deaths and more than 412 million infections worldwide at time of writing. During this period there have been several variants of concern (VoCs), with enhanced transmissibility and/or immune escape (Chen *et al.*, 2021; Davis *et al.*, 2021; Elliott *et al.*, 2021; Graham *et al.*, 2021; Kraemer *et al.*, 2021; O’Toole *et al.*, 2021; Twohig *et al.*, 2022; Wang *et al.*, 2021). Currently, these VoCs are defined by health authorities, World Health Organization and/or by lineages, such as PANGO Lineages (Rambaut *et al.*, 2020) or Nextstrain (Hadfield *et al.*, 2018). These designations are reactive, based on a set of novel mutations must be observed as a distinct clade before being first assigned a lineage, and only then can it be labelled as such in sequencing output and the spread of the variant tracked. There is a clear and pressing need to be able to identify evolutionary ingress of potentially problematic variants as they emerge directly from the sequencing data. Especially as we now approach the endemic stage of the pandemic where transmission levels are high, and surveillance and mitigations may no longer be in place.

To this end, we present Systematic ProtEin AnnotatoR (SPEAR), a tool to flag potential VoCs, highlighting samples that show potentially elevated immune escape and enhanced infectivity at point of sequencing. SPEAR is a lightweight functional genomic surveillance discovery tool, that utilizes information from protein structure, deep mutational scanning (DMS) and computational molecular biophysics to provide comprehensive full protein product annotation for SARS-CoV-2.

## 2 Implementation

SPEAR integrates existing tools with its own internal annotation and QC processes, to ensure that annotation is informative, particularly when dealing with low-quality sequences. A more detailed overview of the implementation is available in Supplementary Data S1 and Figure S1.

### 2.1 Input

SPEAR is written in Python (version 3.10) and utilizes Snakemake (Mölder *et al.*, 2021) for workflow management and parallel job execution. SPEAR is flexible, allowing for single and multi-sample input in the form of either: consensus FASTA sequence, FASTA multiple sequence alignment (MSA) or VCF file(s).

### 2.2 Variant detection

SPEAR aligns consensus input files to SARS-CoV-2 reference genome NC_045512.2 using MUSCLE (Edgar, 2004). Single nucleotide polymorphisms (SNPs) are obtained from the alignment. SPEAR detects indels and multi-nucleotide polymorphisms (MNPs) in the alignment and combines linked events (e.g. SNP followed by deletion) into a single VCF row for accurate amino acid (AA) consequence description.

### 2.3 Quality control

SPEAR integrates several quality control (QC) checks for input samples. All consensus and alignment inputs are checked for unknown base (N) content in the genome (default 50%), which is a sign of poor sequence quality and may bias downstream score estimates. SNPs can also be optionally filtered to remove commonly problematic positions.

SPEAR also provides a Spike protein dropout detection system with user configurable parameters to flag gaps in Spike coverage (>150 bp) due to amplicon dropout, as well as flagging high levels of global N content (>25%), and Ns in the Spike receptor binding domain (RBD) (>12 nt). Dropout detection is critical as missing mutations in Spike cannot be scored and need to be drawn to the user's attention.

### 2.4 Spear annotation

SnpEff (Cingolani *et al.*, 2012) is leveraged to annotate basic variant consequences. Compound HGVS.p format variants are then expanded by SPEAR to individual AA variants, e.g.:

S: p. G142_Y145delinsD to G142D, V143del, Y144del, Y145del

SPEAR utilizes the AA and gene annotations from SnpEff for its downstream functional and structural annotations. The full SnpEff annotation is retained in the final VCF file produced for each sample. SPEAR examines the AA variants in the Spike and evaluates these to show potential increases in immune escape relative to that of the original ‘wild-type’ for the different Barns classes (Barnes *et al.*, 2020) of antibody binding epitopes using DMS data (Dong *et al.*, 2021; Greaney *et al.*, 2021a, b; Starr *et al.*, 2021a, b, c; Tortorici *et al.*, 2021). ACE2 binding is assessed using DMS scores available for the Spike RBD (Starr *et al.*, 2020) which show the likely impact of each mutation, and Vibrational Difference Scores (VDS) (Teruel *et al.*, 2021) which shows the propensity for the open conformational state (that correlates with infectivity). Scoring operates over the RBD (AA: 331–531) for all scores except VDS, which covers a larger region of Spike (AA: 14–913). Descriptions of the scoring system can be found in Supplementary Data S1.

SPEAR also annotates all structural, non-structural and accessory proteins for which structural information is present, including protein–protein interaction interfaces (such as replicase complex subunits and oligomerization), ligand binding and active sites residues as well as domain boundaries, using a manually curated set of annotations derived from protein structures. Example per sample output for BA.1 is found in Supplementary Data S2.

### 2.5 Summary output

SPEAR will provide a per run score summary which sums immune escape and ACE2 interaction scores for each sample. This is the sum of scores for all variants within the sample. A terminal output table of these scores is produced using the Rich python package.

SPEAR produces a HTML report with interactive heatmaps of scores for all samples and metrics as well as sortable tables of mutations and associated scores, built using Plotly and Bootstrap. This report is distributed with all dependencies required for offline viewing. Per sample ORF plots can also be viewed that enable the mutations to be shown against the protein product they reside in along with associated scores. Supplementary Data S3 contain example HTML reports and ORF plots for the Alpha, Delta, Omicron, BA.1, BA.1.1 and BA.2 lineages.

### 2.6 Baseline comparison

SPEAR is distributed with a set of baseline scores for lineages and VoCs which can be selected to compare samples against. The default is to compare to BA.1 (Alpha, Delta, Omicron, BA.1, BA.1.1 and BA.2 can also be selected) or a user provided baseline can be used. Where scores exceed the baseline, these are highlighted in both the HTML and terminal sample summary tables. This enables samples scoring higher for any one metric than the current predominant lineage to be highlighted.

## 3 Application

**Fig. 1.**
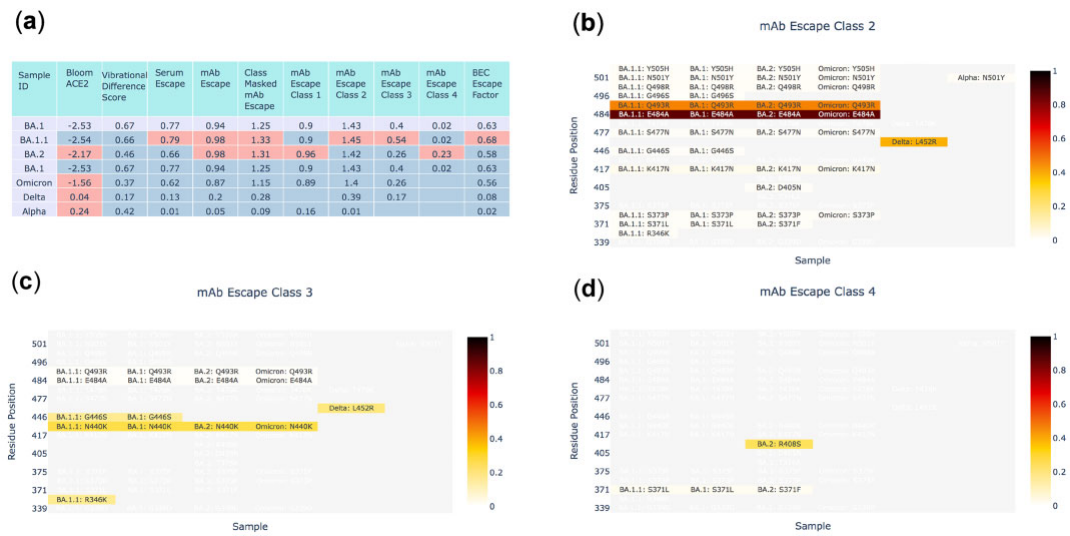
Scoring comparison of mutations in lineages of current and past global interest, taken from the SPEAR summary report. (**a**) Summary score table baselined to compare against BA.1. (**b**) Heatmap of residue mutation scores for class 2 mAb escape in each lineage. Compared to Alpha and Delta, Omicron and it’s sublineages BA.1, BA.1.1 and BA.2 have significantly higher mutational load in Spike RBD contributing to modified ACE2 binding and immune escape scores. The mutation driving increased class 2 escape in BA.1.1 (S: R346K) can be seen to be unique to this lineage. BA.2 can also be seen to be missing S: G446S mutation, leading to an observed reduced class 2 (and class 3) mAb escape score compared to BA.1/BA.1.1. (**c**) Heatmap of class 3 mAb escape. S: R346K stands out as being unique to BA.1.1 and drives an increase in escape in this lineage. (**d**) Heatmap of class 4 mAb escape. The mutation S: R408S drives an increase in class 4 mAb escape in BA.2

SPEAR can be used to identify new sets of mutations within samples of interest from genomic surveillance. The example report baselined to Delta (Supplementary Data S3) highlights this for historical and current lineages/VoCs. Both the heatmaps and scores summary table show that Omicron and its BA sublineages score higher for immune escape than both Alpha and Delta, immediately drawing attention to the emergence of new threats in a surveillance setting (Fig. 1a). Using a report baselined to BA.1 (predominant at time of writing in UK) (Supplementary Data S3), it is apparent that BA.1.1 (a recently designated sublineage) has increased immune escape specifically for epitope classes 2 and 3 (Fig. 1b and c), driven by S: R346K. BA.2 (a Variant Under Investigation) shows increased escape in classes 1 (driven by S: D405N) and 4 (driven by S: R408S, Fig. 1d), but reduced escape in classes 2 and 3 owing to the lack of S: G446S (Fig. 1b and c).

## 4 Conclusion

SPEAR provides rapid assessment of mutations in SARS-CoV-2 samples and can be run without reliance on external web servers. Together with its lightweight implementation, this allows for deployment both in the field and in pathogen surveillance labs worldwide.

## Funding

This work was supported by COG-UK; the Research England’s Expanding Excellence in England (E3) Fund to M.B. and M.C.; and the UK Health Security Agency to M.B. R.J.N. is a member of the Réseau Québécois de Recherche sur les Médicaments (RQRM) and the Quebec Network for Research on Protein Function, Engineering and Applications (PROTEO).

*Conflict of Interest*: none declared.

## Supplementary Material

btac391_Supplementary_Data
