# Supplementary material for: SPEAR: Systematic ProtEin AnnotatoR
Source: Bioinformatics. 2022 Jun 13;38(15):3827–9. doi: 10.1093/bioinformatics/btac391 (PMC9344845; doi:10.1093/bioinformatics/btac391)
Supplement: btac391_Supplementary_Data [file btac391_supplementary_data.zip › Supplemental_S1_RESPONSE.docx]

**SPEAR Detailed Overview**

SPEAR integrates existing tools for alignment and genome annotation together with its own pre-processing and structural/functional annotation tools (Supplementary Figure 1). Initially, consensus inputs can be aligned to reference using either MUSCLE v3.8 or minimap2, specified using --aligner. Minimap2 alignments are converted to pairwise FASTA alignments using GoFasta (reference/sample). FASTA or multiple FASTA input files are pre-filtered to exclude samples that are more than 30% N by default (tuned with --cut-off). SPEAR will quality check and flag any FASTA or multiple FASTA inputs containing: global N percentage (>25%), Spike N percentage (>5%), Spike dropout (contig of N >150nt), and Receptor Binding Domain (RBD) quality (>12nt N content). These QC warnings are displayed in SPEAR outputs in the following format: ! - Spike N contig, ^ - Spike RBD N content, * - Global N percentage, # - Spike N percentage. All cut-offs are user-configurable. By default, SPEAR will filter out the variants occurring in the most 5' 1-55 nucleotides and final 3' end of the genome 29,804-29,903 from all input file types, as sequenced genomes frequently miss this region in addition to it exhibiting a high degree of variability. Additionally, there are known problematic sites in SARS-CoV-2 sequencing, maintained in VCF format by Goldman et al. We expose the ability to filter sites flagged as "mask" in this repository with user defined granularity by invoking --mask-problem-sites and a tag reflecting the type of problematic site to be filtered (or all for all problematic sites) – marking is performed with VCFAnno and filtering with SnpSift.

Following QC, SNPs are obtained in VCF format using UCSC FaToVCF. MNPs and indels are then identified in the resulting VCF by SPEAR internally. Raw SNPs, MNPs and indels (which can also be input to SPEAR directly in VCF format) are annotated using SnpEff and the MN908947.3 database. SPEAR utilises the Gene ID, HGVS.p and Feature type annotations from SnpEff, however, all SnpEff annotation is retained in the final VCF outputs. SPEAR further breaks down the SnpEff annotation of polyprotein ORF1ab into individual protein products: leader protein (nsp1), nsp2, nsp3, nsp4, 3C-like proteinase (nsp5), nsp6, nsp7, nsp8, nsp9, nsp10, RNA-dependent RNA polymerase (RdRp, nsp12), helicase (nsp13), 3'-to-5' exonuclease (nsp14), endoRNAse (nsp15), and 2'-O-ribose methyltransferase (nsp16), allowing for a more granular annotation of structural features in each product.

SPEAR uses several scoring methods to evaluate the likely impact of genomes, as described in Table S1. All scores can be compared to a baseline lineage or user-supplied sample (default BA.2), meaning any scores above the baseline will be highlighted, flagging samples with enhanced immune escape or ACE2 binding. Scoring is supported by annotation of structural features, including region, domains, and residue contacts, along with Barnes antibody class for any mutations in residues in these regions. The file spear_score_summary.tsv contains scores for each sample analysed; its format is detailed in Table S2.

On completion, a terminal table is displayed showing scores for all samples meeting minimum QC requirements, with scores highlighted and compared to the selected baseline. SPEAR outputs annotation in various file formats to provide maximum utility to the end-user, including single and multi-sample VCF format and TSV format summary files. The primary output is the SPEAR report, an interactive HTML summary of all analysed samples. This includes heatmaps and ORF plots of mutations, as well as summary tables, allowing for rapid interrogation of samples for potentially problematic new mutations.


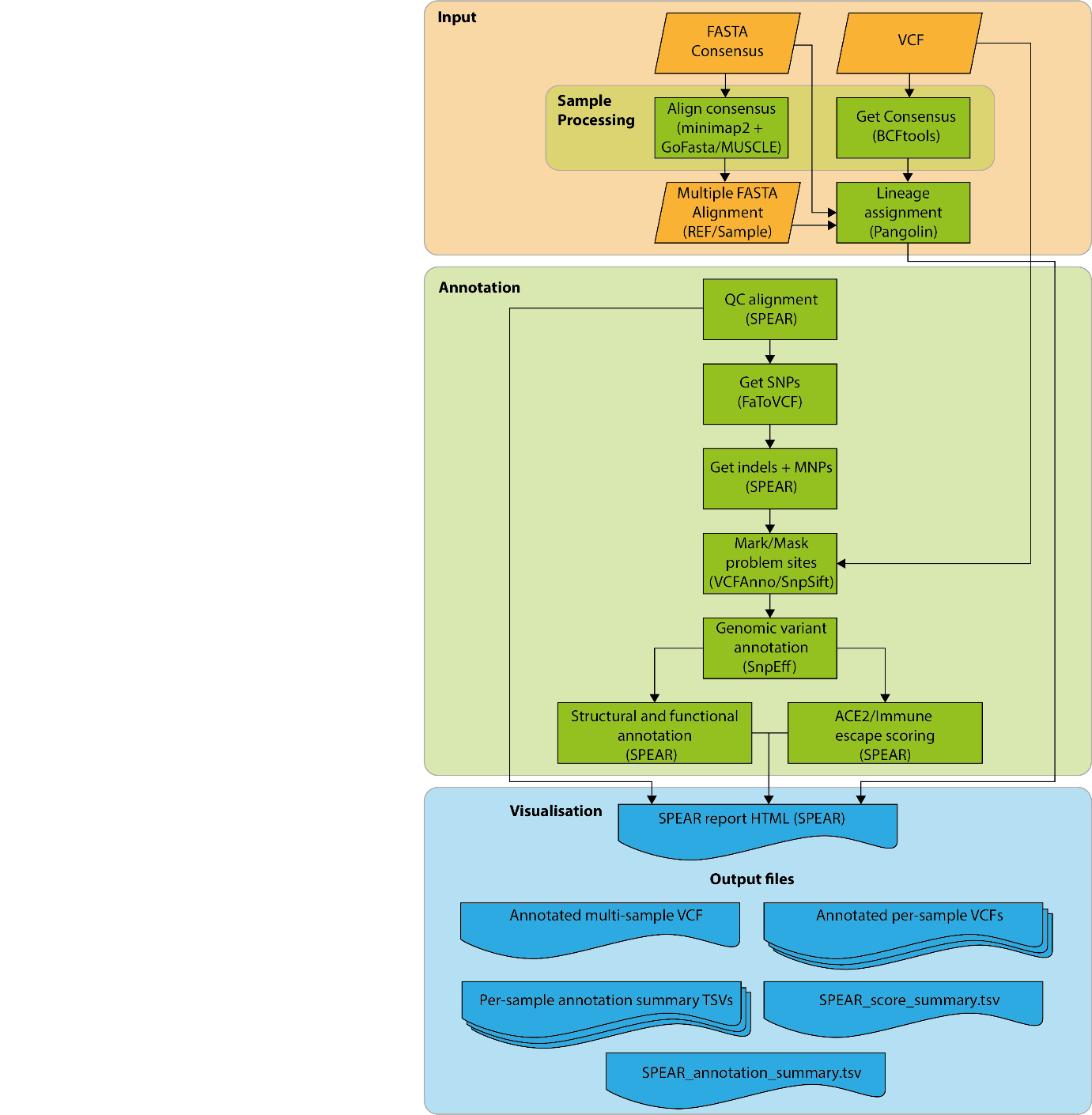


**Figure S1**. Flowchart diagram of the SPEAR pipeline. Inputs to SPEAR can be in three formats: FASTA consensus, multi-FASTA format alignment, or VCF format. SPEAR QC checks input sequences in FASTA or multi-FASTA format for quality characteristics: global N content, Receptor Binding Domain (RBD) N content, and contiguous N sequences, which are highlighted in the final report. Variant annotation is first performed using SnpEff to obtain genomic level information, i.e. gene and variant consequence. This is followed by SPEAR specific annotation of SARS-CoV-2 structural and functional features determined through molecular dynamics, deep mutational scanning data and structural studies. All samples are assigned lineages with Pangolin; for VCF inputs variant calls are first converted into a consensus FASTA sequence. All annotation, lineages and quality control information is summarised in a final SPEAR report, which contains interactive heatmaps, tables and summary information allowing end-users to identify potentially problematic new sequences from their structural/functional characteristics.

**Supplementary S1**

| Column ID | Level | Description |
| --- | --- | --- |
| bloom_ace2 | mutation | ACE2 binding value Δlog10(KD,app) relative to the "wild-type" (WT) [(Starr et al., 2020)](https://app.readcube.com/library/05d6c1ae-b4a4-44db-9a9d-293935e57630/all?uuid=8188813852418683&item_ids=05d6c1ae-b4a4-44db-9a9d-293935e57630:f6ff09c9-7572-4129-ad9e-34ff8a30598f). Higher positive values mean binding is stronger than WT, negative values mean binding is weaker than WT. |
| VDS | mutation | Vibrational Difference Score (VDS) [(Teruel et al., 2021)](https://app.readcube.com/library/05d6c1ae-b4a4-44db-9a9d-293935e57630/all?uuid=2500043368924668&item_ids=05d6c1ae-b4a4-44db-9a9d-293935e57630:6ba8a420-4fdd-4358-bd29-984394b2ca20) positive VDS values suggests mutation stabilises the open state of Spike and/or makes the closed state more flexible, favouring the open conformation relative to the WT. Negative values suggest mutation favours the closed state more than WT. |
| serum_escape | mutation | Mean residue specific serum escape score [(Greaney, Loes, et al., 2021; Dong et al., 2021; Greaney, Starr, Barnes, et al., 2021; Starr, Greaney, Dingens, et al., 2021; Starr, Greaney, Addetia, et al., 2021; Starr, Czudnochowski, et al., 2021; Tortorici et al., 2021)](https://app.readcube.com/library/05d6c1ae-b4a4-44db-9a9d-293935e57630/all?uuid=8905868934936083&item_ids=05d6c1ae-b4a4-44db-9a9d-293935e57630:6980225d-2d71-4886-88f5-635b4e1475e8,05d6c1ae-b4a4-44db-9a9d-293935e57630:46dd2990-37fc-4973-b6f2-0fc8e13143be,05d6c1ae-b4a4-44db-9a9d-293935e57630:6d5f0488-3d97-4190-b3cc-1dff6eb46a9b,05d6c1ae-b4a4-44db-9a9d-293935e57630:8c3c5dc9-655b-4d40-9e7d-e00216732a4a,05d6c1ae-b4a4-44db-9a9d-293935e57630:6a2a9cb6-fc0c-4ef8-9840-f515e84b0d10,05d6c1ae-b4a4-44db-9a9d-293935e57630:8e293972-7bb0-435e-a9e4-e0f12a94e01d,05d6c1ae-b4a4-44db-9a9d-293935e57630:8c4e8af8-e4da-49ef-af19-ef848947991e), larger values indicate more escape, (range 0-1). |
| mAb_escape | mutation | Mean residue specific mAb escape score [(Greaney, Loes, et al., 2021; Dong et al., 2021; Greaney, Starr, Barnes, et al., 2021; Starr, Greaney, Dingens, et al., 2021; Starr, Greaney, Addetia, et al., 2021; Starr, Czudnochowski, et al., 2021; Tortorici et al., 2021)](https://app.readcube.com/library/05d6c1ae-b4a4-44db-9a9d-293935e57630/all?uuid=9307587299617985&item_ids=05d6c1ae-b4a4-44db-9a9d-293935e57630:6980225d-2d71-4886-88f5-635b4e1475e8,05d6c1ae-b4a4-44db-9a9d-293935e57630:46dd2990-37fc-4973-b6f2-0fc8e13143be,05d6c1ae-b4a4-44db-9a9d-293935e57630:6d5f0488-3d97-4190-b3cc-1dff6eb46a9b,05d6c1ae-b4a4-44db-9a9d-293935e57630:8c3c5dc9-655b-4d40-9e7d-e00216732a4a,05d6c1ae-b4a4-44db-9a9d-293935e57630:6a2a9cb6-fc0c-4ef8-9840-f515e84b0d10,05d6c1ae-b4a4-44db-9a9d-293935e57630:8e293972-7bb0-435e-a9e4-e0f12a94e01d,05d6c1ae-b4a4-44db-9a9d-293935e57630:8c4e8af8-e4da-49ef-af19-ef848947991e) from 26 mAbs, larger values indicate more escape, (range 0-1) |
| cm_mAb_escape | mutation | Mean residue specific mAb escape score calculated in a Barns class mask specific way such that the mean is taken only from Barns class mAbs that correspond to the class of residue with mutation. |
| mAb_escape_class_1 | mutation | Mean residue specific mAb escape score from class 1 mAbs only, only applied to residues in Barns class 1 epitope. |
| mAb_escape_class_2 | mutation | Mean residue specific mAb escape score from class 2 mAbs only, only applied to residues in Barns class 2 epitope. |
| mAb_escape_class_3 | mutation | Mean residue specific mAb escape score from class 3 mAbs only, only applied to residues in Barns class 3 epitope. |
| mAb_escape_class_4 | mutation | Mean residue specific mAb escape score from class 4 mAbs only, only applied to residues in Barns class 4 epitope. |
| BEC_RES | residue | Bloom Escape Calculator Residue Escape Score [(Greaney, Starr, and Bloom, 2021)](https://app.readcube.com/library/05d6c1ae-b4a4-44db-9a9d-293935e57630/all?uuid=9488741957278319&item_ids=05d6c1ae-b4a4-44db-9a9d-293935e57630:8998fb85-b907-4c98-9fc2-7fda4fed522b), this residue specific number is generated from the full complement of mutated residues in the sample. Lower values here indicate more antibody escape. |
| BEC_EF | residue | Bloom Escape Calculator Escape Factor [(Greaney, Starr, and Bloom, 2021)](https://app.readcube.com/library/05d6c1ae-b4a4-44db-9a9d-293935e57630/all?uuid=5554479343378783&item_ids=05d6c1ae-b4a4-44db-9a9d-293935e57630:8998fb85-b907-4c98-9fc2-7fda4fed522b), a fraction (0 to 1) of antibodies escaped by mutations at this residue. 0 = no antibodies escaped, 1 = all antibodies escaped. This value is calculated for individual mutations without contribution of other mutated residues. |
| BEC_sample_EF | sample | Bloom Escape Calculator Escape Factor [(Greaney, Starr, and Bloom, 2021)](https://app.readcube.com/library/05d6c1ae-b4a4-44db-9a9d-293935e57630/all?uuid=38602106983785367&item_ids=05d6c1ae-b4a4-44db-9a9d-293935e57630:8998fb85-b907-4c98-9fc2-7fda4fed522b) as BEC_EF but calculated using the full complement of mutated residues in the sample, this score will be the same for every mutated residue in a sample. |

**Table S1**: SPEAR scoring metrics as found in: spear_annotation_summary.tsv

| Column ID | Description |
| --- | --- |
| sample_id | Input sample ID, taken from input file header in .fa, .aln and sample col in .vcf |
| total_variants | Total number of genomic variants at nucleotide level. |
| total_residue_variants | The total number of amino acid changes, here, note insertions are counted as a single event, each deleted residue is counted. |
| consequence_type_variants | Comma separated list of consequence:count present in sample, summary of values in consequence_type from Table 1. |
| region_residues | Amino acid changes as a comma separated list summarised per product:region:count. |
| domain_residues | Amino acid changes as a comma separated list summarised per product:domain:count. |
| ACE2_contact_counts | Total number of mutated amino acids involved in ACE2 contacts. |
| ACE2_contact_score | Sum of contact scores, salt bridges:3, hydrogen-bonds:2, generic residue contact:1. |
| trimer_contact_counts | Total number of mutated amino acids involved in trimer interface contacts, this is specifically for Spike. |
| trimer_contact_score | Sum of contact scores for Spike trimer interface, salt bridges:3, hydrogen-bonds:2, generic residue contact:1. |
| barns_class_variants | Total number of mutated residues per barns class epitope summarised as comma separated list, as class:count additional residues not formally part of epitope but with strong Deep Mutational Scanning (DMS) evidence as having an impact on binding of mAbs of that class are flagged with *. |
| bloom_ACE2_sum | Sum of ACE2 binding value Δlog10(KD,app) relative to the "wild-type" (WT) across all mutated residues[(Starr et al., 2020)](https://app.readcube.com/library/05d6c1ae-b4a4-44db-9a9d-293935e57630/all?uuid=22709516540235297&item_ids=05d6c1ae-b4a4-44db-9a9d-293935e57630:f6ff09c9-7572-4129-ad9e-34ff8a30598f). Higher positive values mean binding is stronger than WT, negative values mean binding is weaker than WT. |
| bloom_ACE2_max | Highest scoring residue for ACE2 value out of all mutated residues. |
| bloom_ACE2_min | Lowest scoring residue for ACE2 value out of all mutated residues. |
| VDS_sum | Sum of all Vibrational Difference Scores (VDS) [(Teruel et al., 2021)](https://app.readcube.com/library/05d6c1ae-b4a4-44db-9a9d-293935e57630/all?uuid=0761588332454064&item_ids=05d6c1ae-b4a4-44db-9a9d-293935e57630:6ba8a420-4fdd-4358-bd29-984394b2ca20), positive VDS values suggest mutation stabilises the open state of Spike and/or makes the closed state more flexible, favouring the open conformation relative to the WT. Negative values suggest mutation favours the closed state more than WT. |
| VDS_max | Highest scoring residue for VDS value out of all mutated residues. |
| VDS_min | Lowest scoring residue for VDS value out of all mutated residues. |
| serum_escape_sum | Sum of mean residue specific serum escape score [(Greaney, Loes, et al., 2021; Dong et al., 2021; Greaney, Starr, Barnes, et al., 2021; Starr, Greaney, Dingens, et al., 2021; Starr, Greaney, Addetia, et al., 2021; Starr, Czudnochowski, et al., 2021; Tortorici et al., 2021)](https://app.readcube.com/library/05d6c1ae-b4a4-44db-9a9d-293935e57630/all?uuid=6598186891417402&item_ids=05d6c1ae-b4a4-44db-9a9d-293935e57630:6980225d-2d71-4886-88f5-635b4e1475e8,05d6c1ae-b4a4-44db-9a9d-293935e57630:46dd2990-37fc-4973-b6f2-0fc8e13143be,05d6c1ae-b4a4-44db-9a9d-293935e57630:6d5f0488-3d97-4190-b3cc-1dff6eb46a9b,05d6c1ae-b4a4-44db-9a9d-293935e57630:8c3c5dc9-655b-4d40-9e7d-e00216732a4a,05d6c1ae-b4a4-44db-9a9d-293935e57630:6a2a9cb6-fc0c-4ef8-9840-f515e84b0d10,05d6c1ae-b4a4-44db-9a9d-293935e57630:8e293972-7bb0-435e-a9e4-e0f12a94e01d,05d6c1ae-b4a4-44db-9a9d-293935e57630:8c4e8af8-e4da-49ef-af19-ef848947991e) across all mutations, larger values indicate more escape. |
| serum_escape_max | Highest scoring residue for serum escape out of all mutated residues. |
| serum_escape_min | Lowest scoring residue for serum escape out of all mutated residues. |
| mAb_escape_all_classes_sum | Sum of mean residue specific mAb escape scores for all mutated residues in sample [(Greaney, Loes, et al., 2021; Dong et al., 2021; Greaney, Starr, Barnes, et al., 2021; Starr, Greaney, Dingens, et al., 2021; Starr, Greaney, Addetia, et al., 2021; Starr, Czudnochowski, et al., 2021; Tortorici et al., 2021)](https://app.readcube.com/library/05d6c1ae-b4a4-44db-9a9d-293935e57630/all?uuid=16254521064419192&item_ids=05d6c1ae-b4a4-44db-9a9d-293935e57630:6980225d-2d71-4886-88f5-635b4e1475e8,05d6c1ae-b4a4-44db-9a9d-293935e57630:46dd2990-37fc-4973-b6f2-0fc8e13143be,05d6c1ae-b4a4-44db-9a9d-293935e57630:6d5f0488-3d97-4190-b3cc-1dff6eb46a9b,05d6c1ae-b4a4-44db-9a9d-293935e57630:8c3c5dc9-655b-4d40-9e7d-e00216732a4a,05d6c1ae-b4a4-44db-9a9d-293935e57630:6a2a9cb6-fc0c-4ef8-9840-f515e84b0d10,05d6c1ae-b4a4-44db-9a9d-293935e57630:8e293972-7bb0-435e-a9e4-e0f12a94e01d,05d6c1ae-b4a4-44db-9a9d-293935e57630:8c4e8af8-e4da-49ef-af19-ef848947991e). |
| mAb_escape_all_classes_max | Highest scoring residue for mAb escape out of all mutated residues. |
| mAb_escape_all_classes_min | Lowest scoring residue for mAb escape out of all mutated residues. |
| mAb_escape_class_1_sum | As above, sum of mean residue specific mAb escape score for all mutated residues from class 1 mAbs only, only applied to residues in Barns class 1 epitope. |
| mAb_escape_class_1_max | Highest scoring residue for class 1 mAb escape out of all mutated residues. |
| mAb_escape_class_1_min | Lowest scoring residue for class 1 mAb escape out of all mutated residues. |
| mAb_escape_class_2_sum | As above, sum of mean residue specific mAb escape score for all mutated residues from class 2 mAbs only, only applied to residues in Barns class 2 epitope. |
| mAb_escape_class_2_max | Highest scoring residue for class 2 mAb escape out of all mutated residues. |
| mAb_escape_class_2_min | Lowest scoring residue for class 1 mAb escape out of all mutated residues. |
| mAb_escape_class_3_sum | As above, sum of mean residue specific mAb escape score for all mutated residues from class 3 mAbs only, only applied to residues in Barns class 3 epitope. |
| mAb_escape_class_3_max | Highest scoring residue for class 3 mAb escape out of all mutated residues. |
| mAb_escape_class_3_min | Lowest scoring residue for class 1 mAb escape out of all mutated residues. |
| mAb_escape_class_4_sum | As above, sum of mean residue specific mAb escape score for all mutated residues from class 4 mAbs only, only applied to residues in Barns class 4 epitope. |
| mAb_escape_class_4_max | Highest scoring residue for class 4 mAb escape out of all mutated residues. |
| mAb_escape_class_4_min | Lowest scoring residue for class 4 mAb escape out of all mutated residues. |
| BEC_EF_sample | Bloom Escape Calculator [(Greaney, Starr, and Bloom, 2021)](https://app.readcube.com/library/05d6c1ae-b4a4-44db-9a9d-293935e57630/all?uuid=5554479343378783&item_ids=05d6c1ae-b4a4-44db-9a9d-293935e57630:8998fb85-b907-4c98-9fc2-7fda4fed522b) Escape Factor a fraction (0 to 1) of antibodies escaped for this sample. 0 = no antibodies escaped, 1 = all antibodies escaped. Calculated using the full complement of changed residues in the whole sample. |

**Table S2**: SPEAR Summary Score fields and descriptions as found in: spear_score_summary.tsv

**References**

[Dong,J. *et al.* (2021) Genetic and structural basis for SARS-CoV-2 variant neutralisation by a two-antibody cocktail. *Nat Microbiol*, **6**, 1233–1244.
Greaney,A.J., Starr,T.N., and Bloom,J.D. (2021) An antibody-escape calculator for mutations to the SARS-CoV-2 receptor-binding domain. *Biorxiv*, 2021.12.04.471236.
Greaney,A.J., Loes,A.N., et al. (2021) Comprehensive mapping of mutations in the SARS-CoV-2 receptor-binding domain that affect recognition by polyclonal human plasma antibodies. *Cell Host Microbe*, **29**, 463-476.e6.
Greaney,A.J., Starr,T.N., Barnes,C.O., et al. (2021) Mapping mutations to the SARS-CoV-2 RBD that escape binding by different classes of antibodies. *Nat Commun*, **12**, 4196.
Starr,T.N., Greaney,A.J., Dingens,A.S., et al. (2021) Complete map of SARS-CoV-2 RBD mutations that escape the monoclonal antibody LY-CoV555 and its cocktail with LY-CoV016. *Cell Reports Medicine*, **2**, 100255.
Starr,T.N. et al. (2020) Deep Mutational Scanning of SARS-CoV-2 Receptor Binding Domain Reveals Constraints on Folding and ACE2 Binding. Cell, **182**, 1295-1310.e20.
Starr,T.N., Greaney,A.J., Addetia,A., et al. (2021) Prospective mapping of viral mutations that escape antibodies used to treat COVID-19. *Science*, **371**, 850–854.
Starr,T.N., Czudnochowski,N., et al. (2021) SARS-CoV-2 RBD antibodies that maximize breadth and resistance to escape. *Nature*, **597**, 97–102.
Teruel,N. et al. (2021) Modelling conformational state dynamics and its role on infection for SARS-CoV-2 Spike protein variants. *Plos Comput Biol*, **17**, e1009286.
Tortorici,M.A. et al. (2021) Broad sarbecovirus neutralization by a human monoclonal antibody. Nature, 597, 103–108.](https://app.readcube.com/library/?style=Bioinformatics)
