# Supplementary material for: SPEAR: Systematic ProtEin AnnotatoR
Source: Bioinformatics. 2022 Jun 13;38(15):3827–9. doi: 10.1093/bioinformatics/btac391 (PMC9344845; doi:10.1093/bioinformatics/btac391)
Supplement: btac391_Supplementary_Data [file btac391_supplementary_data.zip › Supplemental data S3 RESPONSE.zip!/delta_baseline/report.html]

Summary Report

From a total of 6 input samples, 6 passed QC and were annotated by SPEAR. A total of 122 nucleotide variants were identified across all samples, which resulted in 110 AA missense, deletion or insertions which are listed and evaluated below.

Nucleotide Variants

Genomic variants detected across all samples. Sorted by variant count, use dropdown to sort by genomic position.

Amino Acid Variants

Amino acid missense, deletion, or insertion mutations across all samples. Sorted by count of mutation, use dropdown to sort by genomic position.

Residue Score Heatmap

Summary heatmap of all residue positions for which there is at least one mutation across all samples.
Individual sample IDs and mutations are plotted on datapoint, for large samples sets these become visible at higher zoom levels.
Hover text will show z: selected score for sample-residue and sample ID and mutation.
For a description of these scores see Table 3 in the SPEAR README.
For a full screen view of the current plot see here, and for a fullscreen heatmap across all residues (easier comparison between reports), see here.

Per Sample Scores Summary

Summarised scores per sample (sum across sample), cells with values higher than selected baseline are highlighted.
Selected baseline is always shown in the top row, table is sorted by the cm mAb escape all classes sum column by default,
the drop down can be used to sort on other scores. For a description of these scores see Table 4 in the SPEAR README. For a full screen view of this table see here. Source data used to produce this table can be found in the file `spear_score_summary.tsv`  
Quality warnings: ! - Spike N contig (default 150nt) ; ^ - Spike RBD N content (default 12nt) ; \* - Global N percentage (default > half N percentage cutoff) ; # - Spike N percentage (default > 5%)

##### ORF Product Plots

|  |  |  |
| --- | --- | --- |
| Sample ID | Product Plot | AA Mutations |
| Alpha | Alpha Product Plot | 23 || BA.1.1 | BA.1.1 Product Plot | 60 || BA.1 | BA.1 Product Plot | 59 || BA.2 | BA.2 Product Plot | 58 || Delta | Delta Product Plot | 19 || Omicron | Omicron Product Plot | 40 |

Generated On: 2022-02-18

SPEAR Version 0.6.0

SPEAR was developed by Matt Crown and Matt Bashton at Northumbria University UK from work funded by COG-UK. Please post bugs, issue and feature requests on GitHub.
